# The Human Impact of Floods: a Historical Review of Events 1980-2009 and Systematic Literature Review

**DOI:** 10.1371/currents.dis.f4deb457904936b07c09daa98ee8171a

**Published:** 2013-04-16

**Authors:** Shannon Doocy, Amy Daniels, Sarah Murray, Thomas D. Kirsch

**Affiliations:** Johns Hopkins Bloomberg School of Public Health, Baltimore, Maryland, United States; Johns Hopkins Bloomberg School of Public Health, Baltimore, Maryland, United States; Johns Hopkins Bloomberg School of Public Health, Baltimore, Maryland, United States; Johns Hopkins University School of Medicine and Bloomberg School of Public Health, Baltimore, Maryland, United States

## Abstract

Background. Floods are the most common natural disaster and the leading cause of natural disaster fatalities worldwide. Risk of catastrophic losses due to flooding is significant given deforestation and the increasing proximity of large populations to coastal areas, river basins and lakeshores. The objectives of this review were to describe the impact of flood events on human populations in terms of mortality, injury, and displacement and, to the extent possible, identify risk factors associated with these outcomes. This is one of five reviews on the human impact of natural disasters Methods. Data on the impact of floods were compiled using two methods, a historical review of flood events from 1980 to 2009 from multiple databases and a systematic literature review of publications ending in October 2012. Analysis included descriptive statistics, bivariate tests for associations and multinomial logistic regression of flood characteristics and mortality using Stata 11.0. Findings. There were 539,811 deaths (range: 510,941 to 568,680), 361,974 injuries and 2,821,895,005 people affected by floods between 1980 and 2009. Inconsistent reporting suggests this is an underestimate, particularly in terms of the injured and affected populations. The primary cause of flood-related mortality is drowning; in developed countries being in a motor-vehicle and male gender are associated with increased mortality, whereas female gender may be linked to higher mortality in low-income countries. Conclusions. Expanded monitoring of floods, improved mitigation measures, and effective communication with civil authorities and vulnerable populations has the potential to reduce loss of life in future flood events.

## Introduction

Floods are the leading cause of natural disaster deaths worldwide and were responsible for 6.8 million deaths in the 20th century. Asia is the most flood-affected region, accounting for nearly 50% of flood-related fatalities in the last quarter of the 20th century 1 ^,^ 2 ^,^ 3 . The Center for Research on the Epidemiology of Disasters (CRED) defines a flood as “a significant rise of water level in a stream, lake, reservoir or coastal region” 4. More colloquially, flooding is the “presence of water in areas that are usually dry” 1. The events and factors that precipitate flood events are diverse, multifaceted, and interrelated. Weather factors include heavy or sustained precipitation, snowmelts, or storm surges from cyclones whereas important human factors include structural failures of dams and levies, alteration of absorptive land cover with impervious surfaces and inadequate drainage systems. Geographic regions such as coastal areas, river basins and lakeshores are particularly at risk from storms or cyclones that generate high winds and storm surge 5. Environmental/physical land features including soil type, the presence of vegetation, and other drainage basin characteristics also influence flood outcomes 6. Floods transpire on varying timelines, ranging from flash floods with little warning to those that evolve over days or weeks (riverine). Flash floods, characterized by high-velocity flows and short warning times have the highest average mortality rates per event and are responsible for the majority of flood deaths in developed countries 1 ^,^ 3 ^,^ 7. In contrast, riverine floods which are caused by gradual accumulation of heavy rainfall are less likely to cause mortality because of sufficient time for warning and evacuation. Occasionally floods are associated with secondary hazards such as mudslides in mountainous areas.

Recent accelerations in population growth and changes in land use patterns have increased human vulnerability to floods. Harmful impacts of floods include direct mortality and morbidity and indirect displacement and widespread damage of crops, infrastructure and property. Immediate causes of death in floods include drowning and trauma or injury 1 ^,^ 8. Over an extended time period, there may also be increased mortality due to infectious disease 1 ^,^ 9 ^,^ 10 ^,^ 11 . The risks posed by future flood events are significant given population growth, proximities of populations to coastlines, expanded development of coastal areas and flood plains, environmental degradation and climate change 12. The objectives of this review were to describe the impact of floods on the human population, in terms of mortality, injury, and displacement and to identify risk factors associated with these outcomes. This is one of five reviews on the human impact of natural disasters, the others being volcanoes, cyclones, tsunamis, and earthquakes.

## Methods

Data on the impact of flood events were compiled using two methods, a historical review of flood events and a systematic literature review for publications relating to the human impacts of flooding with a focus on mortality, injury, and displacement.

**Historical Event Review**

A historical database of significant floods occurring from 1980 to 2009 was created from publicly available data. Multiple data sources were sought to ensure a complete listing of events, to allow for both human and geophysical factors to be included, and to facilitate cross checking of information between sources. The two primary data sources were CRED International Disaster Database (EM-DAT) 4 and the Dartmouth Flood Observatory (DFO) Global Archive of Large Flood Events database 13. For inclusion in the EM-DAT database, one or more of the following criteria must be fulfilled: 10 or more people killed or injured; 100 people affected; declaration of a state of emergency; or a call for international assistance. The DFO database provides a comprehensive list of flood events recorded by news, governmental, instrumental, and remote sensing sources from 1985 to 2009. Inclusion criteria are: significant damage to structures or agriculture, long intervals since the last similar event, or fatalities. Flooding specifically related to hurricane storm surge and tsunamis were excluded.

Event lists from both databases were downloaded in July 2007 and merged to create a single database; the database was updated in August 2009. The EM-DAT and DFO databases included 2,678 and 2,910 events, reported, respectively, between 1980 and 2009. Both EM-DAT and DFO reported the date and location of the event, the affected region and the number dead. In addition, the number affected, homeless, and total affected (sum of injured, homeless, and affected) were reported by EM-DAT. DFO also reported the number displaced, duration of the event (days), and ‘flood magnitude.’ Flood magnitude is a composite score of flood severity developed by DFO that encompasses damage level, recurrence interval, duration of the flood in days and the area affected 13. For flood impacts reported by EM-DAT, zeroes were treated as missing values because they were used as placeholders and their inclusion in the analysis could contribute to the under estimation of tsunami impacts. The final list included 2,678 events reported by EM-DAT and 2,910 reported by DFO; 1,496 events were reported by both sources yielding a total of 4,093 flood events affecting human populations. See http://www.jhsph.edu/refugee/natural_disasters/_Historical_Event_Review_Overview.html for the database of flood events.

To assess risk factors for flood-related mortality the following categories were used: no deaths (0 deaths), low (1-9 deaths), medium (10- 49 deaths) and high (≥50 deaths). Bivariate tests for associations between flood mortality and the following characteristics were performed using χ^2^ (categorical measures) and ANOVA (continuous measures): decade, region (defined by the World Health Organization (WHO)), income level (World Bank), gross domestic product (GDP), GINI (measure of income inequality), and flood magnitude. All covariates, with the exception of GINI, which was not strongly associated with flood mortality in adjusted analyses, and GDP, which was highly correlated with per capita World Bank income level, were included in the final multinomial logistic regression model to assess the relative risk of mortality at a given level as compared to events with no deaths. All analyses were performed using Stata Statistical Software, Version 11.0 14.

**Systematic Literature Review**

Key word searches in MEDLINE (Ovid Technologies, humans), EMBASE (Elsevier, B.V., humans), SCOPUS (Elsevier B.V., humans), and Web of Knowledge, Web of Science (Thomson Reuters) were performed to identify articles published in July 2007 or earlier that described natural hazards and their impact on human populations. One search was done for all the five natural hazards described in this set of papers. This paper describes the results for cyclones. The systematic review is reported according to the PRISMA guidelines. Key words used to search for natural hazards included*natural hazard(s), natural disaster(s), volcano(s), volcanic, volcanic eruption, seismic event, earthquake(s), cyclone(s), typhoon(s), hurricane(s), tropical storm(s), flood(s), flooding, mudslide(s), tsunami(s), and tidal wave(s)*. Key words included for impact on human populations were *affected, damage(d), injury, injuries, injured, displaced, displacement, refugees, homeless, wounded, wound(s), death(s), mortality, casualty, casualties, killed, died, fatality, fatalities* and had to be used in either the title, abstract or as a subject heading/key word. The search resulted in 2,747 articles from MEDLINE, 3,763 articles from EMBASE, 5,219 articles from SCOPUS, and 2,285 articles from ISI Web of Knowledge. Results from the four databases were combined and duplicates were excluded to yield a total of 9,958 articles.

A multi-stage screening process was used. First, title screening was performed to identify articles that were unrelated to natural disasters or human populations. Each title was screened by two independent reviewers and was retained if either or both reviewers established that inclusion criteria were met. To ensure consistent interpretation of inclusion criteria, percent agreement was assessed across reviewers for a small sample of articles, and title screening began after 80% agreement on inclusion was achieved. A total of 4,873 articles were retained for abstract review. Articles that met one or more of the following criteria were excluded in the abstract screening: language other than English; editorial or opinion letter without research-based findings; related to environmental vulnerability or hazard impact but not human populations; individual case report/study; focus on impact/perceptions of responders; and not related to human or environmental vulnerabilities or impacts of hazards. As with the title screening, 80% overall agreement between reviewers was needed before abstract screening started. Each abstract was screened by two independent reviewers and was retained if either or both established that inclusion criteria were met. Included abstracts were coded for event type, timeframe, region, subject of focus, and vulnerable population focus. A total of 3,687 articles were retained for full article review. Articles discussing the impacts of natural disasters on human populations in terms of mortality, injury, and displacement were prioritized for review. A total of 119 articles on flood events meeting the criteria were retained for full review. Upon full review, 27 articles were retained including 17 that underwent standard data abstraction and 11 that were identified as review articles (Figure 1).

**Overview of the systematic literature review process for floods:**
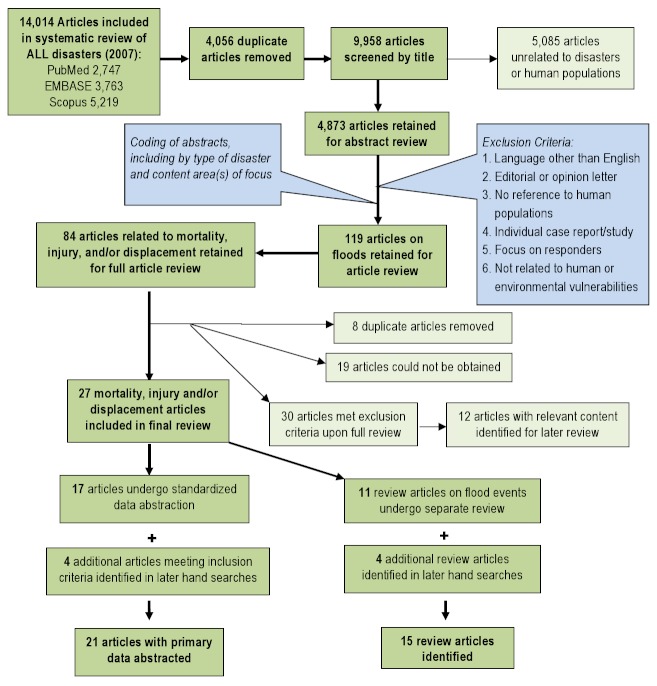

Following the systematic review, a search was conducted to identify relevant articles published after the initial search up to October 2012. This search identified seven additional articles, including three articles with primary data that underwent full review and four review articles. Summaries of abstracted (n=21) and review articles (n=15) are presented in Tables 1 and 2, respectively.

**Table 1: Articles included in the flood systematic literature review relating to mortality and injury* (abstracted, N=21)**:** * Displacement is excluded from the table because no primary data on displacement was collected in only one study, Schnitzler, 2007. ** Additional articles included from the hand searches are Schniztler 2007, Jonkman 2009, Biswas 2010 and Bich 2011.

**Article**	**Event**	**Summary**	**Mortality (n=15)**	**Injury and Morbidity (n=11)**
Janerich, 1981^53^	Hurricane Agnes, 1972, New York, US	Epidemiologic investigation of cancer cases in rural town	Not reported	4 leukemia and lymphoma cases investigated; no increased risk due to flood/environmental hazards identified
Duclos,1991^16^	October 1988,Nimes, France	Surveillance and household survey (n=108) to assess flood health effects	9 drowning deaths reported including two individuals attempting rescues; no risk factors reported	Injuries from surveillance (n=18) included: 3 severe, 3 near drowning, 2 hypothermia, and 10 minor injuries; 6% of 228 survey participants reported minor injuries
Siddique, 1991^17^	Mid-1988, Bangladesh	Record review of health facilities and verbal autopsy	9 of 154 (6%) deaths were directly due to flooding	5% (2,367/46,470) of patients had infected injuries
CDC, 1993^18^	Mid- 1993, Missouri, US	Public health surveillance and medical record review	27 deaths including 21 (78%) direct (drowning); 67% (n=18) of deceased were male	Not reported
CDC, 1993^34^	Summer 1993,Missouri, US	Surveillance of flood-related injuries and illnesses reported at hospitals	Not reported	524 flood-related conditions: 250 injuries (48%) and 233 (45%) illnesses; common injuries were sprains/strains (34%), lacerations (24%), abrasions/contusions (11%)
CDC, 1994^19^	July, 1994,Georgia, US	Record review of flood-related deaths	28 deaths, 96% (n=27) due to drowning; at risk groups were males (71%), adults (86%), and car related (71%)	Not reported
Staes, 1994^20^	Jan 1992,Puerto Rico, US	Descriptive and case-control study of flood mortality	23 deaths; 22 (96%) drowning and 1 (4%) carbon monoxide poisoning; motor vehicles as risk factor	Not reported
Grigg, 1999^28^	July 1997,Colorado, US	Descriptive/historical account	5 deaths reported; 80% were trailer park residents	54 injuries reported; no additional information reported
CDC, 2000^21^	Oct 1998, Texas, US	Public health surveillance and medical record review	31 deaths mostly from drowning (n=24, 77%) and trauma (n=3, 10%); most were male and car related	Not reported
Rashid, 2000^22^	1998, Dhaka Bangladesh	Qualitative survey	918 officially reported flood deaths; qualitative study observed 1200 deaths of which 2% were drownings	Not reported
Ogden, 2001^33^	May 1995,Louisiana, US	Surveillance and record review of disaster-area hospitals and patient visits	Not reported	1855 post-flood injuries, including musculoskeletal (n=791, 46%), lacerations (n=385, 21%), motor vehicle (n=142, 8%), falls (n=134, 7%), and other (n=296, 16%)
Yale, 2003 23	Sept 1999, North Carolina, US	Case-control study of vehicle crashes with drowning	ü 22 deaths reported; males and adults were disproportionately represented	Not reported
Cariappa, 2003^35^	July 2001,Orissa, India	Assessment of flood-related illness/injury in care seekers	Not reported	13% (976/7450) of health facility visits due to injury; males and those 11-40yrs accounted for most injuries
Baxter, 2005^25^>	Jan & Feb1953, UK	Descriptive/historical account	307 deaths due to drowning and exposure; elderly and coastal/poor construction residents were most at risk	Not reported
Gerritsen, 2005^26^	Jan & Feb 1953, The Netherlands	Descriptive review / historical account	1836 deaths; no additional information reported	Not reported
Pradhan, 2007^24^	July 1993, Sarlahi District, Nepal	Household survey in flood affected areas	ü 302 deaths; CMR 7.3/1000; females and young children had greatest risk of death	Not reported
Spencer, 2007^27^	Summer 1977,Pennsylvania, US	Descriptive/historical account	ü 78 deaths; no additional information reported	Not reported
Schnitzler, 2007^36^	August 2002, Saxony, Germany	Telephone survey of flood affected households	ü Not reported	55 (11.7%) of the survey population was injured; risk of injury was increased among those who came into contact with flood water (OR 17.8, 95% CI 17.8– 30.5).
Jonkman, 2009^29^	August 2005,New Orleans	Secondary data analysis of characteristics associated with flood-related mortality following hurricane Katrina	ü Overall mortality percent among exposed was 1%. 853 deaths reported, including 51% male (n=432) and 49% (n=421) female. The majority (85%, 705/829) were among those > 51 yrs of age. In deaths where race was reported (n=819), 55% were African American, 40% white, and 2% other.	Not reported
Biswas, 2010^37^	Summer 2007, Bangladesh	Household survey of child injury in flood-affected areas	ü Not reported	>18% (n=117) children injured were during flood; injuries included 38% lacerations, 22% falls, 21% drowning, 8% road traffic, 6% burns, 5% animal bites.
Bich TH, 2011^54^	October and November 2008, Hanoii, Vietnam	Cross-sectional household survey	ü 2 deaths, no additional information reported	27 injuries, including 18 lacerations/contusions/cuts, 3 fractures, 1 trauma and 5 others. Causes of injuries included falls (16), near-drowning (1) and other (10).

**Table 2: Review articles identified by the systematic review relating to mortality, injury, and displacement in flood events (N=15):** 

**Article**	**Summary**	**Key Findings**
Statistical Bulletin 1974^55^	Review of tornado, flood and hurricane associated mortality in the US from 1965 to 1974	More than 1,200 flood deaths in the United States during the review period with a concentrated in a few large events. 14 major river systems were linked to flood deaths; damage can be mitigated through reforestation, construction of reservoirs and flood walls, diversion, and improved early warning and forecasting systems.
French et al., 1983^45^	Review of National Weather Service flash floods reports from 1969 to 1981 to assess mortality effects of warning systems	Floods were the primary cause of weather-related deaths. There were 1,185 deaths in 32 flash floods with an average of 37 deaths per flood; the highest mortality was associated with dams breaking after heavy rains. Mortality was greater earlier in the study period and twice as many deaths occurred in areas with inadequate warning systems. 93% of deaths were due to drowning, of which 42% were car related.
Avakyan 1999^56^	Review of global flood events from 1997 to 1999 using Dartmouth Flood Observatory data	Damage due to floods increased over time due to more development in flood-affected areas; mapping and regulation of flood hazards zones are necessary to mitigate damage. Globally Bangladesh is the most affected by floods. Number of events, victims, evacuees and damage are reported for each year.
Berz, 2000^39^	Review of the impacts of major floods in the last half of the 20^th^ century and summary of significant floods from 1990 to 1998 from the Munich Re natural event loss database	Floods account for half of all natural disaster deaths; trend analysis suggests the frequency of and damages associated with floods have increased over time. Excluding storm surges, the three most deadly flood events from 1990 to 1998 were in India, Nepal and Bangladesh in 1998–4750 deaths, China in 1998–3656 deaths, and China in 1993-3300 deaths. Explanations for increased mortality include population growth, vulnerability of structures, construction in flood-prone areas, flood protection system failures and changes in environmental conditions.
Beyhun, Altintas & Noji, 2005^31^	Review of the impact of flooding in Turkey from 1970 to 1996	624 floods recorded during study period, including 83 fatal events with 539 deaths. There was an association between deaths and material losses, close to half of flood events occurred in summer months, and 37% of deaths in the Black Sea region.
Guzzetti, 2005^57^	Review of flood and landslide related deaths, missing persons, injuries and homelessness in Italy from 1279 to 2002	50,593 people died, went missing, or were injured in 2,580 flood and landslide events and over 733,000 were displaced. Floods accounted for 38,242 deaths; fatal events were most frequent in the northern Alpine regions and mortality was highest in autumn. Floods were caused by high-intensity or prolonged rainfall, snow melt, overtopping or failure of levees, embankments, or dams, and reservoir mismanagement. Since World War II, landslide has exceeded flood mortality and is comparable to earthquake mortality.
Jonkman & Kelman, 2005^1^	Examination of the causes and circumstances of 247 flood disaster deaths across 13 flood events in Europe and the US	Two-thirds of deaths were due to drowning. Being male and engaging in high risk behavior during flood events were also linked to increased flood mortality. Findings with respect to age-related vulnerability were inconsistent. Authors call for standardization of data collection methodologies across regions and flood types to improve policies and strategies to reduce flood-related death.
Jonkman, 2005^3^	Review of mortality from river floods, flash floods and drainage problems from 1975 to 2002 using the CRED Database	Of all disaster types, floods affect the most people; there were1816 events with 175,000 deaths and 2.2 billion affected from 1975-2002. The deadliest freshwater flood events were Venezuela (1999, 30,000 deaths), Afghanistan (1998, 6,345 deaths), and China (1980, 6,200 deaths). Flash floods resulted in the highest average mortality per event. Average mortality (# fatalities / # affected) was constant across continents while impact magnitude (#s of dead and affected) varied between continents.
Tarhule, 2005^32^	Review of newspaper accounts of rainfall and rain-induced flooding in the Sahel savanna zone of Niger from 1970 to 2000	53 articles reported 79 damaging rainfall and flood events in 47 communities in the Sahel of Niger during the study period; floods destroyed 5,580 houses, killed 18, left 27,289 homeless, and caused over $4 million in damages. Sahel residents attribute floods to five major causes: hydrologic, extreme/unseasonable rainfall, location of affected area, inadequate drainage, and poor construction; cumulative rainfall in the days preceding a heavy rain event is an important predictor of flooding.
Lastoria, 2006^58^	Review of flood deaths and socioeconomic impacts in Italy,1951 to 2003	During study period, ~50% of the flood events resulted in an average of 5 deaths, and about ~10% had >100 deaths. Investigators recommend creating an integrated database to collect more information about flood events in Europe.
Llewellyn, 2006^44^	Review mortality, injury, illness and infectious disease associated with major, recent floods events	In the US, as much as 90% of natural disaster damage (excluding droughts) is caused by floods which cost $3.7 billion annually from 1988 to 1997. There were an average of 110 flood deaths/yr from Between 1940 to 1999, mostly in flash floods and automobile related. Most flood related injuries are mild, and predominantly consist of cuts, lacerations, puncture wounds, and strains/sprains to extremities.
Ahern, 2005^30^	Review of studies of global flood events and assessment of gaps in knowledge relative to reducing public health impact of flooding	Review of 212 epidemiologic studies with detailed findings reported for 36 studies. The majority of flood deaths were due to drowning; deaths due were diarrhea inconclusive though there is some evidence to support increased risk of fecal-oral disease, vector-borne disease and rodent-borne disease. There is a lack of data on frequency of non-fatal flood injury.
Ashley & Ashley, 2008^8^	Review of flood fatalities in the United States from 1959 to 2005	4,585 fatalities over a 47 year period were reported (97.6 deaths/year). No significant increase in flood mortality over time was observed. The majority of flood-related deaths were in flash floods and were motor-vehicle related (63%). Increased risk of flood-related death was observed in individuals ages 10-29 and >60 years.
Jonkman & Vrijling, 2008^49^	Review of mortality attributed to different flood types and presentation of new method for estimating flood related deaths in low-lying areas	Reports on 1883 coastal flood events between 1975 and 2002 resulting in 176,874 deaths and 2.27 billion affected. Mortality by event type was reported as follows: 70 from drainage floods, 392 from river floods and 234 from flash floods. Flood mortality was affected by severity of flood impacts and warning and evacuation. Primary determinants of flood-related death include: lack of warning, inability to reach shelter, building collapse, water depth, rapid rise in water level, water flow velocity, children, and elderly. Applies a new method for estimating loss of life due to floods based on flood characteristics and numbers exposed and mortality among exposed are introduced.
FitzGerald, 2010^50^	Review of flood fatalities in Australia from 1997 to 2008	Estimated 73 flood-related deaths reported from newspapers and historic accounts from 1997 to 2008 in Australia. Most fatalities occurred in the summer months. Drowning deaths were more likely among individuals between the 10-29 and >70 years of age. No difference decline in deaths over time reported. 49% of deaths were motor-vehicle related and 27% were attributed to high risk behavior.

## Results

***Historical Event Review***

Overall, an average of 131 (range 35-287) floods affected human populations annually with the majority (81%) occurred during or after the 1990s. Part of this increase can be explained by improved reporting and by the DFO reporting beginning in 1985. There was great variation in the number of events reported annually between EM-DAT (range 35-213) and DFO (42-235) (Figure 2). While the frequency of flood events increased gradually over time, their impacts on human populations in terms of mortality and affected populations varied greatly between years and were often concentrated around large-scale events (Figure 3). Using the WHO regions the Americas (AMRO) and Western Pacific (WPRO) regions experienced the most flooding events while the fewest were reported in Europe (EURO) (Figure 4). Deaths were overwhelmingly concentrated in South East Asia (SEARO), which accounted for 69% of global flood mortality, though both the Americas (AMRO) and Western Pacific (WPRO) had significant minorities of flood fatalities. The great majority of the flood affected population was in WPRO (59%) and SEARO (35%) of the global total. Overall, the human impacts of floods in Europe, Africa, and the Eastern Mediterranean regions were limited; together the regions accounted for no more than 8% of flood deaths and 4% flood affected populations, respectively. The overall impact of flooding on human populations is summarized in Table 3.

**Reporting of flood events by source and year:**
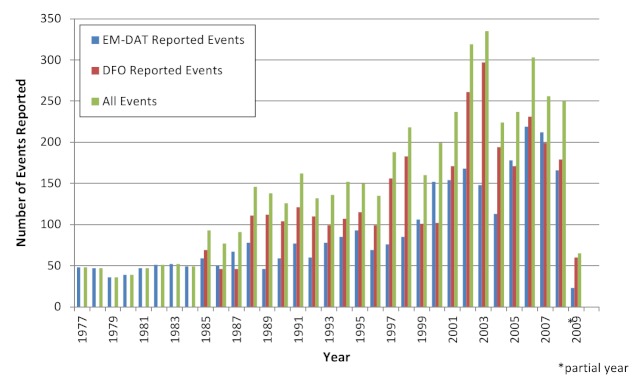

**Flood events affecting human populations by year:**
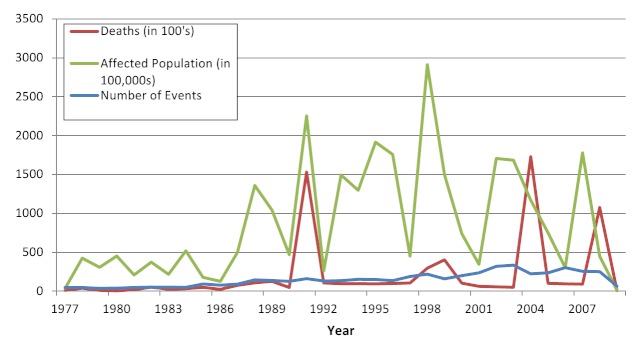

**Regional summary of flood events and their effects on human populations, 1980-2009*:**
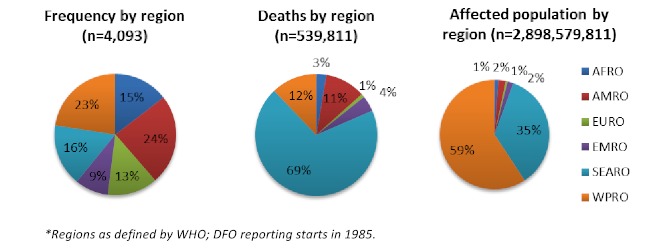

**Table 3: Summary measures for the impact of floods on human populations, 1980-2009 (N=4,093)*:** *Figures are based on the highest reported number of deaths or injuries in an event. Deaths were reported in 4,093 events. Homeless, injured, and total affected populations are reported only by EM-DAT, thus ranges are not presented for overall impact estimates.

***Reported Overall Impact of Flooding Events***					
***Human Consequence***	**# of Events**		**Best Estimate**		**Range**
Deaths	4,093		539,811		510,941-568,680
Injuries	401		362,122		---
Homeless	611		4,580,522		---
Total Affected	2,632		2,898,579,881		---
***Event Summary Statistics***					
***Human Consequence***	**# of Events**		**Median**	**Mean**	**Range**
***Deaths, all events***	**3,960**	**96.8%**	**9**	**135**	**0-138,000**
Reported by EM-DAT	2,646	64.6%	10	74	0-30,000
Reported by DFO	2,732	66.75%	11	166	0-138,000
***Events with deaths***	**2,673**	**65.3%**	**11**	**146**	**1-138,000**
Reported by EM-DAT	2,146	52.4%	10	87	1-30,000
Reported by DFO	1,289	31.5%	13	178	1-138,000
**Injured, all events**	401	9.8%	12.5	904	0-249,378
**Homeless, all events**	611	14.9%	15	7,506	0-2,951,315
**Total Affected, all events**	2,632	64.3%	6,000	1,071,829	0-238,973,000

*Affected Population.* An estimated 2.8 billion people were reported to be affected by flood events between 1980 and 2009, including nearly 4.6 million rendered homeless. However, these figures likely substantially underestimate the true impact of floods on human populations because estimates of the total affected population and the homeless population were reported in only 64.3% (n=2,632) and 14.9% (n=611) of events, respectively. The distribution of the number affected was highly skewed with mean and median affected populations of 1,071,829 and 6,000 per event, respectively, which indicates that the median affected population may better reflect the impact of a typical flood event.

*Mortality and Injury.* When mortality data from the two sources were combined, deaths were reported in 96.8% (n=3,960) of floods since 1980. This figure excludes 13.9% of floods where no information on mortality was reported; if no deaths are presumed and these events are included, deaths occurred in 65.3% (n=2,673) of floods. 539,811 deaths (range: 510,941-568,680) resulting from flood events were reported. For floods where mortality was reported, there was a median of 9 (mean=135; range 0-138,000) deaths per event when using the highest reported death toll. Mortality exceeded 10,000 in only 4 events and 100,000 in two. The two deadliest events occurred in Bangladesh (138,000 deaths in 1991) and Myanmar (100,000 deaths in 2008). Injuries were reported in 401 (9.8%) events, where a total of 361,974 injuries were documented. In events where injuries were reported, there was a median of 12.5 (mean=904: range 1-249,378) per flood event. To estimate the total number of injuries due to flood events, it was presumed that injuries would occur in events where deaths were reported. There were 2,673 floods with fatalities but only 401 (9.8%) with injuries reported. When the median and mean for injuries were applied to the remaining 3,077 events, it was estimated that between 38,463 and 2,717,681 additional unreported flood related injuries may have occurred between 1980 and 2009.

Bivariate associations between country-level characteristics and flood-related mortality from 1980 through 2009 are presented in Table 4. Findings suggests that the proportion of events with high mortality (>50 deaths) have decreased over time. Income level was also significantly associated with flood mortality, where for both low and lower-middle income countries, a greater proportion of events fell in the medium and high death categories as compared to higher income countries. Higher mortality events were concentrated in the South East Asian and Western Pacific regions.

**Table 4: Flood event mortality characteristics, 1980-2009 (N = 4,093):** *GINI coefficient scores for income distribution range from 0 to 100 with 0 representing a perfect equality and 100 perfect inequality.^59^ ** Magnitude is a composite score of flood severity created by DFO that includes flood duration and affected area size, with the following categories: low magnitude,6.0. Flood magnitude is only available for events from 1985 onward.

***Characteristic***	**No deaths (n=706)**	** 1-9 deaths (n=1,378)**	**10-49 deaths (n=1,223)**	**>50 deaths (n=785)**	***P*-value**
***Decade, N (%) ***					
1980	121 (17%)	149 (11%)	212 (17%)	205 (26%)	<.001
1990	191 (27%)	418 (30%)	437 (35%)	317 (40%)	
2000	394 (55%)	811 (58)	574 (45%)	263 (33%)	
***World Bank Development Level, N (%)***					
Low income	172(24%)	263 (20%)	370 (30%)	365 (45%)	<.001
Lower Middle income	164 (23%)	395 (29%)	465 (38%)	328 (41%)	
Upper-middle income	142 (20%)	276 (21%)	219 (18%)	79 (10%)	
High Income	227 (32%)	408 (30%)	176 (14%)	33 (4%)	
***World Health Organization Region, N (%) ***					
Africa	139 (20%)	228 (17%)	157 (13%)	73 (8%)	<.001
Americas	182 (26%)	387 (29%)	293 (24%)	122(15%)	
Eastern Mediterranean	46 (6%)	107 (8%)	147 (12%)	74 (9%)	
European	171 (23%)	246 (18%)	104 (9%)	26 (3%)	
South East Asian	47 (7%)	137 (10%)	229 (19%)	264 (33%)	
Western Pacific	124 (18%)	238 (18%)	299 (24%)	262 (32%)	
**Gross Domestic Product, per capita, mean (SD), (n=4,089)**	14,827 (18,077)	14,330 (17,710)	1,457(12,563)	3,325(6,518)	<.001
GINI,* mean (SD), (n=3,830)	40.2 (7.6)	41.0 (7.7)	41.7 (7.9)	41.3 (7.1)	0.004
Magnitude,** mean (SD), (n=2911)	4.8 (1.2)	4.9 (1.1)	5.3 (1.0)	6.0 (1.1)	<.001

Findings from the adjusted analyses (Table 5) modeling the relative risk of flood related mortality show that all predictors were significantly associated with flood mortality. The relative risk of medium- and high-level mortality events compared to events with no deaths significantly decreased over time. There was also a significant decreased relative risk of mortality in excess of 50 deaths for events in higher income countries compared with lower income country events. Additionally, as magnitude of a flood increased, so did the risk of having high mortality when adjusting for all other predictors. A flood rated as high magnitude as compared to one with low magnitude was associated with an increased relative risk of having high mortality as compared to no mortality (RR=13.20, 95% CI 8.25, 22.11). Caution should be taken when interpreting such findings, however, as magnitude estimates were missing for a large proportion of events, and missing magnitude was associated with the outcome in this study. Regional differences in reported mortality were also supported by the analysis. Higher mortality events were concentrated in the South East Asian and Western Pacific regions, compared to events occurring in the Americas (Southeast Asia RR=3.35, 95 CI: 2.21, 5.72; Western Pacific RR=2.38, 95 CI: 1.62, 3.34).

**Table 5: Multinomial logistic regression results for mortality in flood events, 1980-2009 (N =4,093)*:** * Reference is “no deaths” for all categories (n=743) **see Table 4 notes for definition of flood magnitude

Characteristic	1-9 deaths COR (95% CI)	P- value	10-49 deaths COR (95% CI)	P- value	>50 deaths COR (95% CI)	P-value
**Decade**						
1980	Reference		Reference		Reference	
1990	1.09 (0.87, 1.37)	.426	1.64 (1.29-2.07)	<.001	2.61 (1.99-3.42)	<.001
2000	0.86 (0.64, 1.15)	.313	1.85 (1.39-2.46)	<.001	4,46 (3.22-6.18)	<.001
**World Health Organization Region******						
AMRO	Reference		Reference		Reference	
AFRO	1.09 (0.76-1.55)	.0.62	0.58 (0.41-0.84)	.005	0.35 (0.22-0.56)	<.001
EURO	0.72 (0.54-0.96)	.024	0.45 (0.32-0.63)	<.001	0.31 (0.18-0.52)	<.001
EMRO	1.31 (0.83-2.06)	.240	1.49 (0.95-2.33)	.082	1.31 (0.78-2.21)	.3120
WPRO	0.80(0.59-1.09)	.165	1.22 (0.88-1.67)	.217	2.38(1.62-3.49)	<.001
SEARO	1.61(1.04-2.49)	.032	2.15 (1.40-3.29)	<.001	3.35 (2.21-5.72)	<.001
**World Bank Income Level**						
Low	Reference		Reference		Reference	
Lower middle	152 (1.06-1.92)	0.007	0.99 (0.74-1.34)	.992	0.59 (0.43-0.82)	0.002
Upper middle	1.56 (1.05-2.13)	0.014	0.90 (0.62-1.29)	.576	0.39 (0.24-0.61)	<.001
High	1.16 (0.86-1.71)	0.400	0.29 (0.20-0.42)	<.001	0.05 (0.03-0.08)	<.001
Flood Magnitude Category**						
Low	Reference		Reference		Reference	
Medium Low	1.03 (0.74, 1.44)	.859	1.47 (1.03, 2.10)	.035	1.52 (.95, 2.43)	.0878
Medium High	1.19 (0.85, 1.69)	.310	2.19 (1.50, 3.16)	<.001	3.87 (2.45, 6.10)	<.001
High	0.91 (0.62, 1.35)	.664	2.37 (1.58, 3.55)	<.001	13.20 (8.25, 21.11)	<.001
Missing	0.19 (0.15, 0.25)	<.001	0.32 (0.24, 0.43)	<.001	0.59 (0.40, 0.87)	.007

***Systematic Literature Review***

*Mortality.* Fourteen of the reviewed articles reported mortality data including ten that provided information on direct or indirect causes of mortality and/or risk factors for flood-related deaths (Table 6) 15 ^,^ 16 ^,^ 17 ^,^ 18 ^,^ 19 ^,^ 20 ^,^ 21 ^,^ 22 ^,^ 23 ^,^ 24 ^,^ 25 ^,^ 26 ^,^ 27 ^,^ 28 . Most articles provided some information about the distribution of deaths across population subgroups (i.e. gender, age) and/or an individual’s location at the time of the event; seven of these ten articles reported on floods in the United States. Nearly all articles reporting cause of death cited drowning as the most frequent cause of death 1 ^,^ 15 ^,^ 18 ^,^ 19 ^,^ 20 ^,^ 22 ^,^ 29 . Cumulatively, drowning accounted for 75% of deaths; other causes of death included falls, electrocution, heart attack, hypothermia, trauma, snake bites, and carbon monoxide poisoning.

**Table 6: Primary research articles describing flood related deaths and risk factors for flood mortality (N=10):** *excludes 1150 deaths from diarrhea and other possibly deaths reported during the 4 month period surrounding the event

**Article**	**Country & Year**	**Flood Related Deaths**			**By Cause**		**By Sex**		**By Age**	**Vehicle Related**
		Total	Direct	Indirect	Drowning	Other Causes	Males	Female		
Duclos,1991 16	France, 1988	9	9 (100%)	0 (0%)	9 (100%)	0 (0%)	Not reported		Not reported	Not reported
CDC, 1993^18^	USA, 1993	27	21 (78%)	6 (22%)	21 (78%)	2 (7%) electrocution2 (7%) vehicle accident 2 (7%) cardiac arrest	18 (67%)	9 (33%)	Average age = 38(range 9-88)	13 (48%)
CDC,1994^19^	USA, 1994	28	27 (96%)	1 (4%)	27 (96%)	1 (4%) other	20 (71%)	8 (29%)	Average age = 31(range 2-84)	20 (71%)
Staes,1994^20^	USA, 1992	23	22 (96%)	1 (4%)	22 (96%)	1 (4%) carbon monoxide poisoning	10 (43%)	13 (57%)	16 (70%) ≥ 16 yrs	20 (87%)
Grigg, 1999^28^	USA, 1997	5	5 (100%)	0 (0%)	Not reported		5 (100%)	0 (0%)	All adults	Not reported
CDC, 2000^21^	USA, 1998	31	29 (94%)	2 (6%)	24 (77%)	3 (10%) trauma1 (3%) hypothermia1 (3%) cardiac arrest2 (6%) other	20 (65%)	11 (35%)	Median age = 38(range 2-83)	22 (71%)
Rashid, 2000^22^	Bangladesh, 1998	50*	Not reported		24 (48%)	21 (42%) electrocution 5 (10%) snake bites	Not reported		Children accounted for 92% (22/24) of drownings	Not reported
Yale, 2003^23^	USA, 1999	22	22 (100%)	0 (0%)	22 (100%)	0 (0%)	17 (77%)	5 (23%)	21 (95%) adults	22 (100%)
Pradhan, 2007^24^	Nepal, 1992	302	Not reported		Not reported		126 (42%)	176 (58%)	164 (54%) children138 (46%) adults	Not reported
Jonkman et al., 2009^29^	USA, 2005	853	Not reported		Not reported		432 (51%)	421 (49%)	705 (85%) older than 51 yrs, 60% over 65 yrs	Not reported
**Totals**		**447**	**135 (93%)**	**10 (7%)**	**125 (75%)**	**42 (25%)**	**639 (50%)**	**643 (50%)**	**--- **	**97 (74%)**

All studies in the United States examined mortality related to motor vehicles and found an increased risk of mortality among individuals in motor vehicles during the event, of all deaths 74% were motor vehicle related 17 ^,^ 18 ^,^ 19 ^,^ 20 . This compares to a motor vehicle related death rate of 63% in a recent review of US flood fatalities between 1959 and 2005 7. Higher proportions of deaths among males (64%) were consistently observed in the United States, except for Puerto Rico where 57% (13/23) of flood related fatalities were female and hurricane Katrina where deaths evenly divided between the sexes (51% male, 49% female) 16 ^,^ 18 ^,^ 19 ^,^ 20 ^,^ 28 . In contrast, the one article describing flood mortality in the less developed country of Nepal found that females of all age groups faced increased mortality risk and 58% of all deaths were women 23 Other factors found to be associated with flood-related mortality included storm course/time storm hit landfall 19 ^,^ 22 summer months 17 ^,^ 30, low socioeconomic status 23, poor housing construction^16^ ^,^ 23 ^,^ 24 ^,^ 31 and timing of warning messages 19 ^,^ 22.

*Injury and Displacement.* Injury or morbidity data were reported in ten of the 18 included articles, of which nine provided information on injury type and/or risk factors 15 ^,^ 16 ^,^ 24 ^,^ 32 ^,^ 33 ^,^ 34 ^,^ 35 ^,^ 36 ^,^ 54 . The majority of flood-related injuries are minor. The two studies that captured a large number of injuries, both in the United States, found that musculoskeletal injuries were most common (46% and 34%), followed by lacerations (21% and 24%). Other flood-related injuries included abrasions and contusions, motor vehicle related injuries, and falls 33 ^,^ 34 ^,^ 54. In less developed settings, increased incidence of snake bites and fires were also cited as causes of injury or death 2 ^,^ 36. Among care seekers in flood-affected areas of Bangladesh 5.1% of wounds were infected. Another review suggested that the proportion of survivors requiring medical attention is less than 2% 2. A distribution of injuries across population subgroups was reported by only one study in India which found that injuries were more common in males (67% vs. 33%), that the 11-40 year age group comprised 68% of the injured, and that those age 50 and above accounted for 18% of flood deaths 34. Seven articles reported displacement or evacuation figures however none described risk factors associated with flood-related displacement 15 ^,^ 17 ^,^ 21 ^,^ 24 ^,^ 25 ^,^ 35 ^,^ 37 .

## Discussion

**Main findings**

In the past 30 years approximately 2.8 billion people have been affected by floods with 4.5 million left homeless, at approximately 540,000 deaths and 360,000 injuries, excluding an estimated 38,000 to 2.7 million injuries that went unrecorded. While the mortality estimate presented in this study is consistent with the range of estimates presented in other studies 1 ^,^ 38, approximations of numbers injured and displaced are likely gross underestimates of the true values given the infrequency with which figures are reported. Floods events with high levels of mortality are relatively rare: despite their increasing frequency, there were only four events with >10,000 deaths and 58 events with >1000 deaths between 1977 and 2009. A slight decrease in the average number of fatalities per event was observed which is in keeping with broader natural disaster trends that show an increase in the size of the affected population and a decrease in the average number of deaths per event 4. Higher numbers of fatalities were reported in flash floods than river floods, however, river floods affected larger populations and land areas 3 ^,^ 7. Lower mortality rates in river floods can mostly be attributed to their slower onset allowing for longer time for warning and evacuation 3 ^,^ 39. The widespread use of effective early warning methods for hydrological events has likely contributed declining flood mortality.

Findings from the historical event review are consistent with previous observations that flood mortality varies by region, economic development level, and the severity of the event 12 ^,^ 40. The majority of flood-related deaths are concentrated in less developed and heavily populated countries, with Southeast Asia and the Western Pacific region experiencing the highest risk of flood-related deaths. Flood mortality rates are relatively similar across continents, but Asian floods kill and affect more people because they affect substantially larger areas with larger populations 3. At the country level, lower GDP per capita was linked to higher mortality, which is in keeping with the established relationship between poverty and increased disaster risk 41. Human and social vulnerabilities and inequalities, urbanization, population density, terrain and geo-physical characteristics and variation in the frequency and precipitating causes of floods by region are also factors that contribute flood risk levels 3 ^,^ 6 ^,^ 12 ^,^ 42 . Temporal changes and development trends have also contributed to changing influences of some of these factors over time 42. Economic development increases the risk of disaster-related economic losses however improved emergency preparedness, response, and coping capacity may reduce disaster vulnerability 3. That countries with greater resources are able to better predict and respond to impending flood events suggests that building systems and capacity to detect and respond to floods in less developed countries should be a priority 40.

Causes of and risks for flood-related mortality and injury identified in the systematic literature review are consistent with previous reviews on the human impact of flooding 1 ^,^ 29 ^,^ 43 ^,^ 44 . In comparison, a recent review of 13 flood events in Europe and the United States found that 68% of deaths were due to drowning, 12% trauma, 6% heart attack, 4% fire, 3% electrocution, 1% carbon monoxide poisoning, and 7% other/unknown 1. Studies reporting the gender breakdown for flood-related deaths, most of which are accounts of flood events in the United States, consistently show a greater proportion of males as compared with female deaths. These observations are aligned with previous studies, including a review of flood events in Europe and the US which estimated that males account for 70% of flood related deaths 1 ^,^ 44 ^,^ 45 ^,^ 46 . While limited to only a few countries, these findings suggest there may be increased mortality risk for males in more developed settings and for females in less developed countries 23 ^,^ 47 . An increased risk of death in younger and older populations was also observed which is consistent with broader natural disaster mortality trends 7 ^,^ 45 ^,^ 46 ^,^ 48 ^,^ 49 . In Nepal, children had the highest crude mortality rates of all age groups and were nearly twice as likely to die in the flood as their same-sex parent 23. However, recent reviews of age-specific risk for flood mortality have been inconclusive because attempts to aggregate data were hampered by high proportions of deaths where age is unreported 1. While the prevailing notion is that women and children are more vulnerable in disasters 50, there is a paucity of research in less developed countries where the majority of flood deaths occur. Future research on the human impacts of floods should focus on these less developed settings, most notably Asia where flood deaths are concentrated, with the aim of identifying the most at-risk and vulnerable population sub-groups to better target early warning and preparedness efforts.

The ecological nature of the study of event characteristics did not allow for an examination of specific factors within a country or region that may be associated with increased mortality following a flood event. Population density in coastal regions, which are particularly vulnerable to flooding, is twice of the world’s average population density and many of the world’s coasts are becoming increasingly urbanized 51. Currently, 50.6% of the world’s population lives in urban settings; by 2050 this figure is projected to increase to 70% with the majority of urbanization occurring in less developed regions of Asia and Africa 52. Unabated urbanization and land use changes, high concentrations of poor and marginalized populations, and a lack of regulations and preparedness efforts are factors that will likely contribute to an increasing impact of floods in the future 38. From the natural hazard perspective, climate change is also likely to contribute to future increases in flooding. Increased frequency of intense rainfall, as a result of higher temperatures and intensified convection will likely lead to a rise in extreme rainfall events, more flash floods and urban flooding due to excessive storm water. Additionally, sea level rise and increasing storm frequency will lead to additional storm surges in coastal areas while seasonal changes, notably warmer winters, will contribute more broadly to increased precipitation and flood risk 38. Together, changes in socioeconomic, demographic, physical terrain features and climatologic factors suggests that floods will become more frequent and have greater effects on human populations in the coming decades.

Given that flood losses are likely to increase in future years, increased attention to flood prevention and mitigation strategies is necessary. To date, early warning systems have been an effective mechanism for reducing the impact of floods 38, however, they are not ubiquitous and should be prioritized in less developed countries with large at-risk populations and high frequencies of flooding. It is important that messaging and targeted communication strategies accompany early warnings so that the population understands the impending risk and can respond appropriately. Many flood fatalities are associated with risk-taking behaviors, thus messages to avoid entering flood waters and to curtail risky activities in all stages of the event may be successful in reducing flood fatalities 1. Additional, improved land use planning and regulation of development can mitigate flood impacts. Studies on the relationships between flood losses, natural hazard characteristics, and societal and demographic vulnerability factors can aid in informing and prioritizing flood prevention and mitigation strategies. Finally, comparisons of the effectiveness of different policies and mitigation strategies can inform future strategy and policy actions and ensure they are appropriate in specific contexts.

***Limitations***

The effects of flood events are the subject of gross approximations and aggregations that have a great deal of imprecision. The availability and quality of data has likely increased and improved over time and the use multiple data sources increased reporting. However, in many events deaths are unknown or unrecorded; for other outcomes such as injured and affected, reporting frequency is even lower which likely contributes to a substantial underestimation of the impacts of flood events on human populations. While available data is sufficient for a cursory analysis of global flood impacts and trends, improved reporting of flood outcomes, including the development of national systems capable of more accurately reporting mortality and injury would be beneficial. Regarding the measures used in this study, our multivariable model included a broad classification of income level according to the World Bank, as opposed to GDP. While we believe GDP to be a more precise measure of wealth, it was nonetheless excluded in the analysis because we did not obtain GDP estimates that were time specific to each event. Inconsistencies and errors were common in data files from different sources, and in some cases inclusion criteria were not ideal for the purposes of this review, which created a challenge in reconciling event lists. For example, the 2004 Asian tsunami was classified as a flood by Dartmouth but not by EM-DAT; this event was ultimately removed from the data set, however, it represented the highest mortality event in the study period, which has potentially important implications for analysis. Consistent definitions and categorization of events across sources such as that initiated by EM-DAT in 2007 would be useful for streamlining future analysis and comparing the impacts of different types of flood events. Other principal limitations of the literature review are 1) that an in-depth quality analysis of all reviewed articles was not undertaken, and 2) the fact that only English language publications were included which likely contributed to incomplete coverage of studies published in other languages originating from low and middle income countries.

## Conclusions

Interpretation of flood fatality data is challenging given the occurrence of occasional extreme events, temporal trends and the completeness and accuracy of available data. The continuing evolution of socio-demographic factors such as population growth, urbanization, land use change, and disaster warning systems and response capacities also influences trends. Between 1980 and 2009 there were an estimated 539,811 deaths (range 510,941 -568,584) and 361,974 injuries attributed to floods; a total of nearly 2.8 billion people were affected by floods during this timeframe. The primary cause of flood-related mortality was drowning. In developed countries being in a motor-vehicle at the time of a flood event and male gender were associated with increased mortality risk. Female gender may be linked to higher mortality risk in low-income countries. Both older and younger population sub-groups also face an increased mortality risk. The impact of floods on humans in terms of mortality, injury, and affected populations, presented here is a minimum estimate because information for many flood events is either unknown or unreported.

Data from the past quarter of a century suggest that floods have exacted a significant toll on the human population when compared to other natural disasters, particularly in terms of the size of affected populations. However, human vulnerability to floods is increasing, in large part due to population growth, urbanization, land use change, and climatological factors associated with an increase in extreme rainfall events. In the future, the frequency and impact of floods on human populations can be expected to increase. Additional attention to preparedness and mitigation strategies, particularly in less developed countries, where the majority of floods occur, and in Asia, a region disproportionately affected by floods, can lessen the impact of future flood events.

## Competing Interest

The authors have declared that no competing interests exist.

## Correspondence

Shannon Doocy, Johns Hopkins Bloomberg School of Public Health, 615 N. Wolfe St, Suite E8132, Baltimore, MD 21230. Tel: 410-502-2628. Fax: 410-614-1419. Email: sdoocy@jhsph.edu.

## Appendix 1

### PRISMA Checklist

Download PDF
